# Potential and limitations of inferring ecosystem photosynthetic capacity from leaf functional traits

**DOI:** 10.1002/ece3.2479

**Published:** 2016-09-22

**Authors:** Talie Musavi, Mirco Migliavacca, Martine Janet van de Weg, Jens Kattge, Georg Wohlfahrt, Peter M. van Bodegom, Markus Reichstein, Michael Bahn, Arnaud Carrara, Tomas F. Domingues, Michael Gavazzi, Damiano Gianelle, Cristina Gimeno, André Granier, Carsten Gruening, Kateřina Havránková, Mathias Herbst, Charmaine Hrynkiw, Aram Kalhori, Thomas Kaminski, Katja Klumpp, Pasi Kolari, Bernard Longdoz, Stefano Minerbi, Leonardo Montagnani, Eddy Moors, Walter C. Oechel, Peter B. Reich, Shani Rohatyn, Alessandra Rossi, Eyal Rotenberg, Andrej Varlagin, Matthew Wilkinson, Christian Wirth, Miguel D. Mahecha

**Affiliations:** ^1^ Max Planck Institute for Biogeochemistry Jena Germany; ^2^ Amsterdam Global Change Institute VU University Amsterdam Amsterdam The Netherlands; ^3^ German Centre for Integrative Biodiversity Research (iDiv) Halle‐Jena‐Leipzig Leipzig Germany; ^4^ Institute of Ecology University of Innsbruck Innsbruck Austria; ^5^ Institute of Environmental Sciences Leiden University Leiden The Netherlands; ^6^ Mediterranean Center for Environmental Studies (Foundation CEAM) Valencia Spain; ^7^ FFCLRP‐USP Ribeirão Preto Brasil; ^8^ Eastern Forest Environmental Threat Assessment Center USDA Forest Service Raleigh NC USA; ^9^ Department of Sustainable Agro‐Ecosystems and Bioresources Research and Innovation Center, Fondazione Edmund Mach Trento Italy; ^10^ Foxlab Joint CNR‐FEM Initiative Trento Italy; ^11^ UMR 1137 Ecologie et Ecophysiologie Forestierès INRA Champenoux France; ^12^ European Commission Joint Research Centre Institute for Environment and Sustainability Ispra Italy; ^13^ Department of Matters and Energy Fluxes Global Change Research Institute CAS Brno Czech Republic; ^14^ Johann Heinrich von Thünen Institute Federal Research Institute for Rural Areas, Forestry and Fisheries Braunschweig Germany; ^15^ National Hydrology Research Centre (NHRC) Saskatoon Saskatchewan Canada; ^16^ Department of Biology San Diego State University San Diego CA USA; ^17^ The Inversion Lab Hamburg Germany; ^18^ INRA, Grassland Ecosystem Research (UR874) Clermont Ferrand France; ^19^ Department of Physics University of Helsinki Helsinki Finland; ^20^ Provincia Autonoma di Bolzano Servizi Forestali Bolzano Italy; ^21^ Faculty of Science and Technology Free University of Bolzano Bolzano Italy; ^22^ Alterra Green World Research Wageningen The Netherlands; ^23^ Department of Environment, Earth and Ecosystems The Open University Milton Keynes UK; ^24^ Department of Forest Resources University of Minnesota Twin Cities Saint Paul MN USA; ^25^ Hawkesbury Institute for the Environment Western Sydney University Penrith New South Wales Australia; ^26^ Soil and Water Department Faculty of Agricultural, Food and Environmental Quality Sciences The Hebrew University of Jerusalem Rehovot Israel; ^27^ Department of Earth and Planetary Sciences Weizmann Institute of Science Rehovot Israel; ^28^ A.N. Severtsov Institute of Ecology and Evolution Russian Academy of Sciences Moscow Russia; ^29^ Environmental and Human Sciences Division Forest Research Alice Holt Lodge Farnham, Surrey UK; ^30^ Institute of Special Botany and Functional Biodiversity University of Leipzig Leipzig Germany

**Keywords:** ecosystem functional property, eddy covariance, FLUXNET, interannual variability, photosynthetic capacity, plant traits, spatiotemporal variability, TRY database

## Abstract

The aim of this study was to systematically analyze the potential and limitations of using plant functional trait observations from global databases versus in situ data to improve our understanding of vegetation impacts on ecosystem functional properties (EFPs). Using ecosystem photosynthetic capacity as an example, we first provide an objective approach to derive robust EFP estimates from gross primary productivity (GPP) obtained from eddy covariance flux measurements. Second, we investigate the impact of synchronizing EFPs and plant functional traits in time and space to evaluate their relationships, and the extent to which we can benefit from global plant trait databases to explain the variability of ecosystem photosynthetic capacity. Finally, we identify a set of plant functional traits controlling ecosystem photosynthetic capacity at selected sites. Suitable estimates of the ecosystem photosynthetic capacity can be derived from light response curve of GPP responding to radiation (photosynthetically active radiation or absorbed photosynthetically active radiation). Although the effect of climate is minimized in these calculations, the estimates indicate substantial interannual variation of the photosynthetic capacity, even after removing site‐years with confounding factors like disturbance such as fire events. The relationships between foliar nitrogen concentration and ecosystem photosynthetic capacity are tighter when both of the measurements are synchronized in space and time. When using multiple plant traits simultaneously as predictors for ecosystem photosynthetic capacity variation, the combination of leaf carbon to nitrogen ratio with leaf phosphorus content explains the variance of ecosystem photosynthetic capacity best (adjusted *R* ^2^ = 0.55). Overall, this study provides an objective approach to identify links between leaf level traits and canopy level processes and highlights the relevance of the dynamic nature of ecosystems. Synchronizing measurements of eddy covariance fluxes and plant traits in time and space is shown to be highly relevant to better understand the importance of intra‐ and interspecific trait variation on ecosystem functioning.

## 1 Introduction

Accurate predictions of land–atmosphere feedbacks under climate change require an in‐depth understanding of how climatic and other environmental controls on ecosystem functioning are mediated by vegetation characteristics, diversity, and structure (Bonan, 2008). Eddy covariance (EC) measurements of carbon dioxide (CO_2_), water, and energy fluxes are widely employed to monitor ecosystem processes and functions (Baldocchi et al., 2001). The increased number of EC flux sites contributing to the FLUXNET network allows for monitoring ecosystem processes and responses to environmental conditions for different ecosystems and time scales (Baldocchi, 2008). In many applications, both in terrestrial biosphere models and in experimental analyses, the characteristics and structure of the vegetation are given by plant functional types (PFTs), which represent a grouping of functionally similar plant types (Lavorel, Mcintyre, Landsberg, & Forbes, 1997). However, plant traits and model parameters derived from EC data can be highly variable within PFTs and species (Alton, 2011; Groenendijk et al., 2011; Kattge et al., 2011; Reichstein, Bahn, Mahecha, Kattge, & Baldocchi, 2014). Vegetation characteristics and the variation therein are assumed to be determined by the abundance and traits of the respective plant species (Garnier et al., 2004; Lavorel & Garnier, 2002). Therefore, both modeling (Pappas, Fatichi, & Burlando, 2016; Van Bodegom et al., 2012; Verheijen, Aerts, Bonisch, Kattge, & Van Bodegom, 2015) and observational efforts (Meng et al., 2015) increasingly aim to account for the variation of traits within and between PFTs, in order to better understand the relationship between vegetation characteristics and ecosystem functioning. Most efforts so far have focused on specific regions (e.g., Ollinger et al., 2008) and have not systematically analyzed the importance of spatiotemporal variation in traits and ecosystem functional variables for their relationship. Plant traits contribute to different ecosystem processes where our knowledge is often limited. Furthermore, efforts have mostly focused on leaf nitrogen as a functional trait (in relation to ecosystem productivity, e.g., Kattge, Knorr, Raddatz, & Wirth, 2009), whereas other plant traits could also be suitable candidates. Foliar phosphorus, for example, improves the model prediction of carbon fluxes as reported by Mercado et al. (2011), Goll et al. (2012), and Yang, Thornton, Ricciuto, and Post (2014).

The short‐term (half‐hourly to daily) variability of carbon fluxes measured with the EC technique is controlled by meteorological, environmental conditions (Richardson, Hollinger, Aber, Ollinger, & Braswell, 2007), and endogenous plant controls (De Dios et al., 2012). In contrast, biotic responses (e.g., temporal variability in plant abundance and traits) seem to be more important than environmental variation for long‐term (e.g., annual) variation of fluxes (Richardson et al., 2007; Stoy et al., 2009). Evaluating the relationship between plant traits and eddy covariance fluxes is not straight forward because the former is usually measured only a couple of times per year (mostly during the growing season), whereas the latter is measured continuously at half‐hourly intervals. It is possible to derive so‐called ecosystem functional properties (EFP) from EC measurements, a concept recently introduced to characterize the long‐term patterns underlying carbon, water, and energy fluxes (Musavi et al., 2015; Reichstein et al., 2014).

The EFPs are ecosystem properties related to physical and ecohydrological parameters relevant for land surface–atmosphere interactions (Reichstein et al., 2014) and are assumed to be affected by vegetation characteristics. Analogous to leaf level ecophysiological characteristics, such as carboxylation capacity (Vc_max_), EFPs are less variable in time than the fluxes themselves, which makes them a suitable quantity to be linked to plant functional traits (Musavi et al., 2015; Reichstein et al., 2014). Therefore, EFPs can be used to characterize long‐term variation in key process characteristics, such as ecosystem photosynthetic capacity and respiration rates under standardized environmental conditions, or they can represent the sensitivity of processes to temperature and light availability (for a more detailed collection; see Table 1, Musavi et al., 2015). Deriving EFP estimates from EC fluxes is not trivial, because they should represent intrinsic ecophysiological properties of the ecosystem; effects of short‐term meteorological conditions on functional responses should be factored out.

**Table 1:** Definitions of ecosystem photosynthetic capacity estimated using light response curve

Ecosystem photosynthetic capacity	Radiation	Definition
GPP_sat_	PAR	GPP at light saturation using PAR as driving radiation and 2110 μmol m^−2^ s^−1^ as saturating light
GPP_sat.structure_	APAR	GPP at light saturation using APAR as driving radiation and 2000 μmol m^−2^ s^−1^ as saturating light
*A*_max_	PAR	Light saturated GPP—parameter of Equation (1) with PAR as driving radiation
*A*_max.sructure_	APAR	Light saturated GPP—parameter of Equation (1) but with APAR as driving radiation
GPP_cum_	PAR	Integral of the light curve GPP up to the saturation point 2110 μmol m^−2^ s^−1^ of PAR
GPP_cum.structure_	APAR	Integral of the light curve GPP up to the saturation point 2000 μmol m^−2^ s^−1^ of PAR

In the column “Radiation,” the independent variable used in Equation (1) is reported.

Another constraint for testing the links between plant traits and EFPs is that so far, measurements of plant functional traits have not yet been carried out systematically at FLUXNET sites. Consequently, a number of studies linking plant traits and EFPs using a wide range of ecosystems are few (e.g., Kergoat, Lafont, Arneth, Le Dantec, & Saugier, 2008). Although plant trait data from FLUXNET sites are currently limited, the global database of plant traits—TRY (Kattge et al., 2011)—facilitates the identification of many different traits for most of the plant species present at FLUXNET sites, which could potentially help testing such relationships. However, the use of trait values derived from such broadscale databases may suffer from inaccuracies, when trait values for a particular site deviate from those reported in databases, which may hamper deducing the patterns of plant traits influences on EFPs. Hence, it is important to test the potentials and limitations of using plant functional traits derived from a global database (e.g., TRY) versus in situ measurements obtained from the sites to infer the impact of plant traits on ecosystem processes derived from EC flux data. We still do not know how temporal and spatial variations in both EFPs and plant functional traits are linked. Likewise, the uncertainties of the relationship between EFPs to plant functional traits related to the temporal dynamics of both ecosystem functioning and traits have not been evaluated before. This is the first time to our knowledge that the relationship between an EFP (here ecosystem photosynthetic capacity) derived from EC CO_2_ fluxes and plant traits and the associated uncertainties have been systematically investigated for spatiotemporal variation and the relevance of synchronized observations. Using ecosystem photosynthetic capacity as an example for an EFP derived from selected FLUXNET sites, the goals of this study were as follows:

1. Providing an objective approach to characterize ecosystem photosynthetic capacity from different estimates of gross primary productivity (GPP) derived from EC measurements.
2. Assessing how relaxing the time‐space synchronization of ecosystem photosynthetic capacity estimates and plant functional trait measurements introduces uncertainty to their relationships (with a particular focus on leaf nitrogen content per leaf mass).
3. Identifying (a set of) plant traits that control the spatial variability (i.e., across sites) of ecosystem photosynthetic capacity.

## 2 Materials and Methods

The overall methodological approach consisted of comparing different ways to estimate ecosystem photosynthetic capacity at each FLUXNET site. Ecosystem photosynthetic capacity is an EFP related to the photosynthetic processes at ecosystem scale. It is computable from estimates of GPP from EC, incoming photosynthetically active radiation (PAR) and the fraction of absorbed photosynthetically active radiation (FAPAR) retrieved from remote sensing. Given the attempt to characterize properties related to long‐term variation of ecosystem function that are not affected by short‐term meteorological variability, the ecosystem photosynthetic capacity estimates with the least interannual variation (IAV) were assumed as the most appropriate to characterize the EFP. These estimates of ecosystem photosynthetic capacity were correlated with leaf nitrogen content per leaf mass (N) measured in situ or derived from the TRY database to identify the relevance of time and space synchronizing measurements of EC data and plant traits. Finally, ecosystem photosynthetic capacity was correlated with a suite of other photosynthesis‐related plant traits to identify those that control its spatial (i.e., across site) variability.

### 2.1 Eddy covariance flux measurements

The analysis used data from the FLUXNET La Thuile database (Baldocchi, 2008), referred hereafter as “La Thuile.” Very dry sites and forest site‐years with disturbances (i.e., forest thinning, harvesting, and planting) were removed opting for optimal conditions to avoid confounding factors. For the remaining dataset, 20 sites responded to a request for providing leaf traits sampled in 2011/2012 (for some sites, trait measurements from previous years were used) and the flux data from the year of sampling. Depending on the site, different years of flux data were available in the La Thuile database in addition to the fluxes from the sampling year 2011/2012.

To characterize ecosystem photosynthetic capacity, we used half‐hourly values of GPP (μmol CO_2_ m^−2^ s^−1^) and the corresponding PAR (μmol m^−2^ s^−1^). The GPP values were computed using the commonly used algorithm for flux partitioning, which is based on the extrapolation of nighttime net ecosystem exchange measurements using an ecosystem respiration model based on air temperature (Reichstein et al., 2005). As PAR was not always available at the selected sites, we derived PAR by multiplying global incoming shortwave radiation (Rg, Wm^−2^) by 2.11 (Britton & Dodd, 1976).

Only GPP data derived from measured net ecosystem exchange were used for the analysis and gap‐filled values were omitted. In addition, only daytime GPP data were used (Rg > 10 Wm^−2^). For each site‐year, we estimated the number of days with more than 80% gaps in half‐hourly net ecosystem exchange measurements during the period from April to September. Site‐years with more than 25% of such days were excluded.

### 2.2 MODIS TIP‐FAPAR and leaf area index (LAI)—vegetation structure

**Figure 1:**
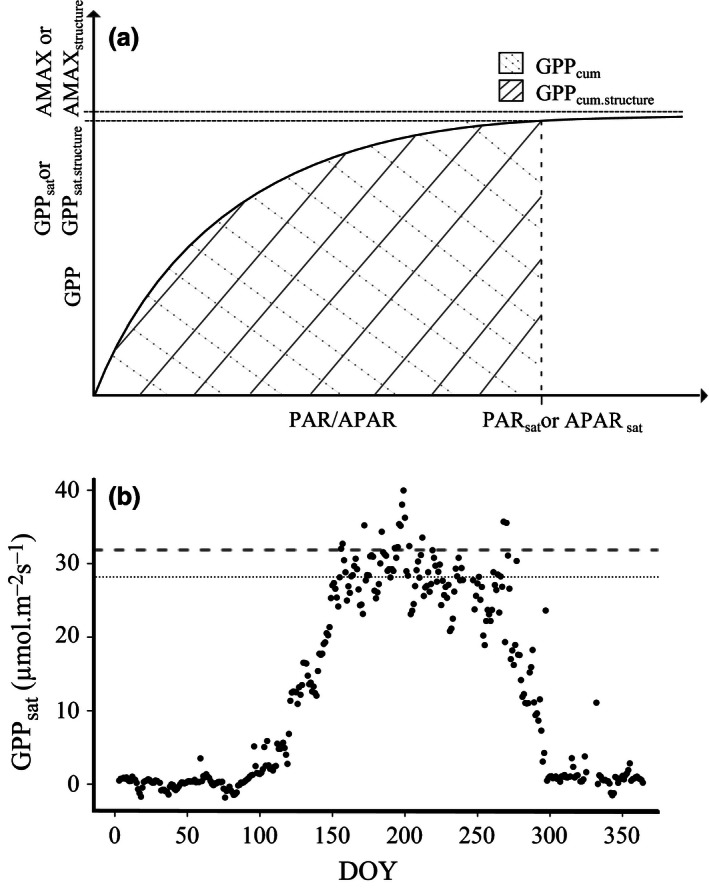
(a) Conceptual figure of the different estimates of ecosystem functional property (EFP) related to ecosystem photosynthetic capacity. Light response curves are fitted using GPP flux and PAR or APAR according to Table 1. (b) Time series of GPP_sat_ for 1 year. Higher values of GPP_sat_ occur during the growing season (usually around mid‐spring to end‐summer). For this study, we use the 90th percentile as the maximum GPP_sat_ of each year, which is indicated with the dashed line. For comparison the 60th percentile of GPP_sat_ is indicated with the dotted line

For the selected sites, estimates of FAPAR and LAI (see Pinty et al., 2011a,b) derived at 1 km spatial resolution by the JRC‐TIP (Pinty et al., 2007) from the MODIS broadband visible and near‐infrared surface albedo products (Schaaf et al., 2002) were used to quantify the vegetation phenology and changes in the structure of the ecosystem with 16‐day temporal resolution (Musavi et al., 2015; Figure 1). We used the FAPAR time series of the pixels where the towers of FLUXNET sites were located. To fill gaps in FAPAR and LAI, we performed a distance correlation between the time series of all pixels around the central pixel for each flux site (Szekely, Rizzo, & Bakirov, 2007). We subsequently chose pixels with a correlation of *r* > .75 with the central pixel. Afterward, we used the data of those pixels to fill the gaps in the central pixel, prioritizing the pixels with highest correlation. In case where gaps remained after this procedure, we used a spatiotemporal gap‐filling approach for the remaining gaps (v. Buttlar, Zscheischler, & Mahecha, 2014). To derive daily time series of FAPAR, a smoothing spline approach was used to derive daily time series of FAPAR (see also Filippa et al., 2016; Migliavacca et al., 2011). FAPAR was then used to compute half‐hourly APAR (absorbed photosynthetic active radiation) values (μmol m^−2^ s^−1^). Annual maximum LAI was derived using the 90^th^ percentile of the satellite retrieved estimates of LAI from JRC‐TIP of the same year of sampling (Pinty et al.*,* 2011a).

### 2.3 Plant functional trait collection—vegetation characteristics

Plant traits known to be relevant for photosynthesis at ecosystem scale, specifically leaf nutrient content and stoichiometry of the nutrients, were determined (Sardans & Penuelas, 2012): leaf nitrogen content per dry mass (N_mass_ or per 100 g leaf dry mass‐ N%), leaf nitrogen content per leaf area (N_area_, g/m^2^), leaf phosphorus content per leaf dry mass (P_mass_, mg/g) and per leaf area (P_area_, g/m^2^), leaf carbon content per leaf dry mass (C, mg/g), leaf C/N ratio (C/N, g/g), leaf stable isotope concentration (δ^13^C), and specific leaf area, (SLA, mm/mg).

In situ leaf samples from the selected sites were collected in the period 2011–2012 (except for two sites in 2003 and in 2004). The leaf sampling protocol was based on “Protocols for Vegetation Sampling and Data Submission” of the terrestrial carbon observations panel of the global terrestrial observing system (Law et al., 2008). Samples were collected from the dominant species present in the footprint of the flux towers (defined by the site's principal investigator). Depending on accessibility, multiple individuals per species were sampled. Sampling was carried out mostly at peak growing season on fully developed and nondamaged leaves and from different levels of the canopy (top, middle, and bottom, representing fully sunlit and shaded leaves). For forest sites, the understory vegetation was not sampled.

After grinding the dried leaves, total carbon and nitrogen concentrations were determined by dry combustion with an elemental analyzer (Perkin Elmer 2400 Series II). Phosphorus concentrations were determined by digesting ground leaf material in 37% HCl: 65% HNO_3_. Phosphorus was subsequently measured colorimetrically at 880 nm after a reaction with molybdenum blue. δ^13^C was determined by an elemental analyzer (NC2500, ThemoQuest Italia, Rodana, Italy) coupled online to a stable isotope ratio mass spectrometer (Deltaplus, ThermoFinnigan, Bremen, Germany). Leaf area was calculated with the ImageJ freeware (http://rsb.info.nih.gov/ij/).

Species abundance information was collected for each site, or if not available (e.g., for one tropical forest site), all species were considered equally abundant. Abundance information for each species was used to calculate the community weighted means (CWM, Garnier et al., 2004) of the different plant traits considered in the analysis: foliar N, P, and C concentration of leaves, SLA, and δ^13^C. Plant trait data were also extracted from the TRY global database (Kattge et al., 2011). Species mean values were calculated from the observed plant trait values included in TRY, which were subsequently used to compute CWM trait values at each site. TRY data used in this study based on the following references: Atkin, Westbeek, Cambridge, Lambers, & Pons, 1997; Bahn et al., 1999; Campbell et al., 2007; Cavender‐Bares, Keen, & Miles, 2006; Coomes, Heathcote, Godfrey, Shepherd, & Sack, 2008; Cornelissen, 1996; Cornelissen et al., 2003; Cornelissen, Diez, & Hunt, 1996; Cornelissen et al., 2004; Cornwell et al., 2008; Craine et al., 2009; Craine, Lee, Bond, Williams, & Johnson, 2005; Diaz et al., 2004; Freschet, Cornelissen, Van Logtestijn, & Aerts, 2010; Fyllas et al., 2009; Garnier et al., 2007; Han, Fang, Guo, & Zhang, 2005; Hickler, 1999; Kattge et al., 2011, 2009; Kazakou, Vile, Shipley, Gallet, & Garnier, 2006; Kerkhoff, Fagan, Elser, & Enquist, 2006; Kleyer et al., 2008; Laughlin, Leppert, Moore, & Sieg, 2010; Louault, Pillar, Aufrere, Garnier, & Soussana, 2005; Loveys et al., 2003; Medlyn et al., 1999; Messier, Mcgill, & Lechowicz, 2010; Meziane & Shipley, 1999; Niinemets, 2001; Ogaya & Penuelas, 2003; Onoda et al., 2011; Ordonez et al., 2010; Poorter, Niinemets, Poorter, Wright, & Villar, 2009; Poschlod, Kleyer, Jackel, Dannemann, & Tackenberg, 2003; Quested et al., 2003; Reich, Oleksyn, & Wright, 2009; Reich et al., 2008; Sack, Cowan, Jaikumar, & Holbrook, 2003; Sack, Melcher, Liu, Middleton, & Pardee, 2006; Shipley, 1995, 2002; Shipley & Vu, 2002; Vile, 2005; White, Thornton, Running, & Nemani, 2000; Willis et al., 2010; Wright et al., 2007, 2004, 2010.

### 2.4 Estimates of ecosystem photosynthetic capacity

To estimate the ecosystem photosynthetic capacity, we used ecosystem level light response curves, using half‐hourly GPP estimates and a variety of radiation data. The resulting six different formulations of ecosystem photosynthetic capacity estimates are reported in Table 1 and described in the following.

We fitted nonrectangular hyperbolic light response curves (Gilmanov et al., 2003):

(1) $$\text{GPP}=\frac{1}{2\theta }\left(\alpha Q+A_{\max}-\sqrt{{\left(\alpha Q+A_{\max}\right)}^{2}-4\alpha A_{\max}\theta Q}\right)$$

where **α** is the initial slope of the light response curve, **θ** is the curvature parameter (ranging from 0 to 1), *A*_max_ is the plateau of the light response curve, GPP is the half‐hourly GPP values, *Q* is the incoming radiation used to drive the model. Specifically, two different estimates of radiation were used (PAR, and APAR): In the estimation of the EFPs, APAR was used to account for seasonal and across‐site variations in canopy structure (e.g., LAI) as it stands for the amount of light that is absorbed by the leaves of the ecosystem.

The ecosystem photosynthetic capacity values were estimated using a 5‐day moving window. The parameters of the light response curves were estimated and attributed to the day at the center of the window (Figure 1a). The parameters were estimated by minimizing the model observation residual sum of square with the quasi‐Newton optimization method that allows box constraints (Byrd, Lu, Nocedal, & Zhu, 1995). To this purpose, we used the *optim* function implemented in R (http://CRAN.R-project.org/). For comparison, a Michaelis–Menten‐based light response curve (Hollinger et al., 2004) was used. Results were comparable with the nonrectangular hyperbolic light response curve (data not shown).

Each light response curve fitting was used to derive the *A*_max_ parameter, the value of GPP at light saturation and the integral of the light response curve at light saturation (Falge et al., 2001). For light saturation, we defined a threshold of Rg of 1,000 Wm^2^ (corresponding to PAR of 2,110 μmol m^−2^ s^−1^) (see also Jacobs et al., 2007). This resulted in six different estimates describing ecosystem photosynthetic capacity: (1) *A*_max_: parameter of the Equation (1); (2) *A*_max.structure_: parameter of Equation (1) but with APAR as driving radiation to account for canopy structure; (3) GPP_sat_: GPP at light saturation using PAR as driving radiation (4) GPP_sat.structure_: as GPP_sat_ but with APAR as radiation variable; (5) GPP_cum_: integral of the fitted light response until light saturation; and (6) GPP_cum.structure_: as GPP_sat_ but using APAR as radiation until light saturation (Figure 1a, Table 1).

A time series of daily values of *A*_max_, *A*_*max*.structure_, GPP_sat_, GPP_sat.structure_, GPP_cum_, and GPP_cum.structure_ was then derived for each year. In Figure 1b, GPP_sat_ is shown as an example. Daily parameters were retained for further analysis only if the *R* ^2^ of the fit of light response curve was higher than 0.6. In this way, we first retain parameters estimated when the performance of the fitting is good, and second, we retain data only in the active growing season as the *R* ^2^ of the model fit was typically higher than 0.6 only within the growing season (Fig. S1).

To extract the corresponding annual ecosystem photosynthetic capacity for each site‐year, maximum and different percentiles (90th to 60th) of the time series of the estimated parameters were computed. Finally, the coefficient of variation (CV, Everitt, 1998) of the annual ecosystem photosynthetic capacity estimates was computed for each site. For example, at each site, we computed the annual value for GPP_sat_ (i.e., 90th percentile of GPP_sat_ daily time series). The CV was subsequently computed as the standard deviation of annual GPP_sat_ of all years available, divided by the mean annual GPP_sat_ for all years available at the respective site (CV GPP_sat_). The CV was used as a measure of the interannual variability (IAV) of the ecosystem photosynthetic capacity estimates. Low IAV (i.e., the lowest CV) was used as criteria to identify the most appropriate estimates to characterize the ecosystem photosynthetic capacity at each site. This was repeated for both ecosystem photosynthetic capacity estimates with and without the effect of canopy structure included (i.e., using PAR and APAR, respectively). This comparison was made using sites with at least 5 years of data. The average of annual ecosystem photosynthetic capacity of the selected estimates was used to relate to leaf functional traits.

### 2.5 Relationship between ecosystem photosynthetic capacity and leaf nitrogen concentration

This study evaluates the relevance of synchronizing measurements of plant functional traits and EFPs in space and time for joint analyses. We analyzed the relationship between the best estimates for ecosystem photosynthetic capacity selected as described above, and CWM of plant traits, for example, N%. N% is chosen here, as the relationship between N% and photosynthetic processes is well established (e.g., Field & Mooney, 1986; Reich, Walters, & Ellsworth, 1997) at the leaf scale and to a lesser extent at ecosystem scale (e.g., Kergoat et al., 2008; Ollinger et al., 2008). The relationship with other traits is included in the supporting information (Fig. S2). Three different combinations of synchronizing ecosystem photosynthetic capacity and N% were tested:

(1) Ecosystem photosynthetic capacity derived from the La Thuile database and species N% derived from TRY (no synchronization in space and time); (2) ecosystem photosynthetic capacity derived from the La Thuile database and the N% sampled at the FLUXNET sites (in situ, synchronization in space); (3) ecosystem photosynthetic capacity derived for the same year of trait sampling and N% in situ (synchronization in space and time).

For each combination of ecosystem photosynthetic capacity and N%, the slope and *R* ^2^ of the linear regression were determined. Distance correlation was computed as well, as it accounts for nonlinear relationships (Szekely et al., 2007). In order to evaluate the predictive capacity of the selected model, a leave‐one‐out cross‐validation was performed. Modeling efficiency (EF; Loague & Green, 1991) and relative root mean square error (RRMSE) were computed to test the performances of the relationships. An analysis of covariance (ANCOVA) was conducted to statistically test the differences of regression slopes in the three relationships. In addition, to assess the significance of canopy structure in the relationship of plant traits and ecosystem photosynthetic capacity, we evaluated the information that LAI, representing the canopy structure, provides to the relation of N% and photosynthetic capacity estimated using GPP and PAR.

### 2.6 Identifying plant functional traits controlling ecosystem photosynthetic capacity

Because the functional relationship between plant traits, their interactions and photosynthetic capacity is not yet completely defined (Sardans & Penuelas, 2012), a purely data‐driven approach was used (Golub, 2010). To identify the main explanatory variables (plant functional traits and LAI) of ecosystem photosynthetic capacity, we used a stepwise multiple regression for variable selection based on the Akaike's information criterion (AIC; Yamashita, Yamashita, & Kamimura, 2007). Plant traits used in this context include N%, N_area_, P_mass_ and P_area_, C, δ^13^C, and SLA. We allowed the variables (traits and LAI) to be raised to the half and second power and also included the logarithm and ratios of all predictors to account for nonlinear relationships and interactions as well.

## 3 Results

### 3.1 Identifying robust estimates to characterize ecosystem photosynthetic capacity

**Figure 2:**
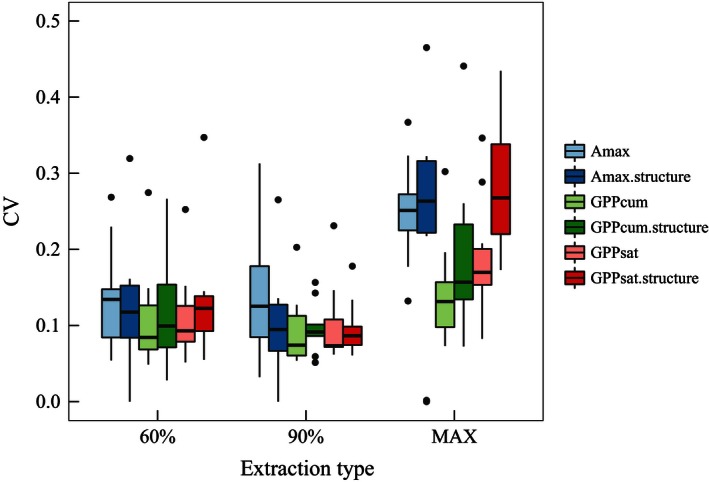
Comparison of mean and ranges of the different estimates of ecosystem photosynthetic capacity and different annual extractions. CV denotes the coefficient of variation (standard deviation/mean), which was calculated for every site. The results are based on sites with at least 5 years of available estimates (AT‐Neu, DE‐Hai, FI‐Hyy, FR‐Hes, IL‐Yat, IT‐MBo, IT‐Ren, IT‐SRo, NL‐Loo, RU‐Fyo). The lines across the box indicate the mean CV values and lower and upper boxes show the 25th and 75th percentiles. The lines on the ending of the boxes range from the maximum to minimum values. CV can be used to quantify the interannual variability of the estimates (small range and low average denote low interannual variability). For explanations of the ecosystem photosynthetic capacity estimates described in the legend, see Table 1

Among the different percentiles that were used for the extraction of annual ecosystem photosynthetic capacity estimates, the 90th percentile was the one that minimized the CV (i.e., the IAV) of most estimators (Figure 2). The maximum annual values showed the highest IAVs and therefore were not considered appropriate estimates of ecosystem photosynthetic capacity. The use of the 60th percentile for the extractions showed slightly higher IAV than the 90th percentile. Other percentiles such as 85, 80, 75, and 70 were also tested and had similar results to the 60 percentile (data not shown). However, considering that we were interested in the annual maximum photosynthetic rates the 90th percentile of the different parameters was selected for further analyses.

**Figure 3:**
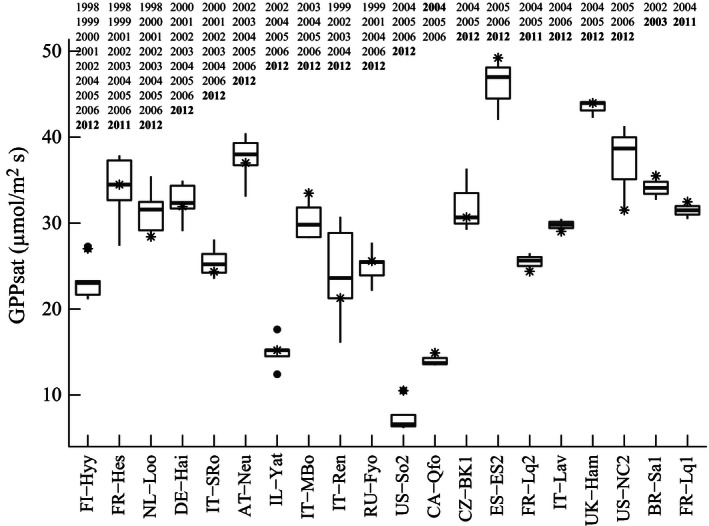
Boxplots of annual GPP_sat_ values derived from the La Thuile database for each FLUXNET site. The line across the boxplot shows the mean GPP_sat_ for each site, and the lower and upper boxes show the 25th and 75th percentiles of GPP_sat_. The stars denote GPP_sat_ values of the respective sites in the year of in situ plant trait measurements (bold years)

Among the different estimators for ecosystem photosynthetic capacity (Table 1), *A*_max_ and *A*_max.structure_ had the highest IAV regardless of how they were extracted annually. GPP_cum_ and GPP_sat_ had the lowest IAV, even though a detailed analysis revealed a substantial IAV for both estimators at some La Thuile sites (Figure 3). While GPP_cum_ is related to the whole growing season, GPP_sat_ is related mostly to the peak of growing season. However, GPP_cum_ and GPP_sat_ were strongly correlated (Table S1). GPP_cum.structure_ and GPP_sat.structure_, accounting for canopy structure, showed slightly higher IAV than GPP_cum_ and GPP_sat_. As we aimed at developing a method to derive maximum ecosystem photosynthetic capacity robust to meteorological variability, we assessed the impact of excluding from the analysis site‐years with documented extreme events, such as the heat wave of 2003 in Europe (Fig. S3). Removing the year 2003 from the European site‐years did not change the results (Fig. S4). In addition, the estimated parameters, for example, GPP_sat_ were not strongly linked to climate variables (Fig. S8).

We concluded that the 90th percentile of GPP_cum_ or GPP_sat_ parameters of nonrectangular hyperbolic light response curves (either with or without structural information included) was an appropriate approach to characterize ecosystem photosynthetic capacity.

### 3.2 Relationship between ecosystem photosynthetic capacity and plant functional traits

**Figure 4:**
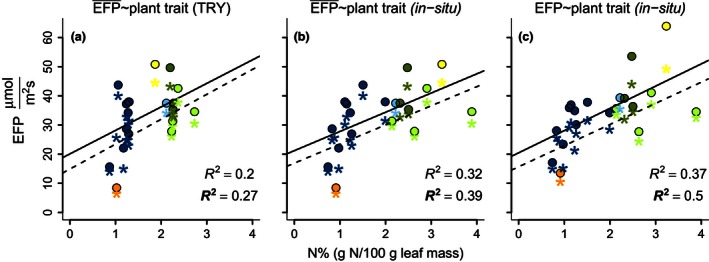
Relationship between a) GPP_sat_ and GPP_sat.structure_ extracted from La Thuile and N% from TRY, b) GPP_sat_ and GPP_sat.structure_ from La Thuile and N% in situ, c) GPP_sat_ and GPP_sat.structure_ derived from the same year of the trait sampling and N% in situ. Y‐axes are ecosystem photosynthetic capacity as an example of an EFP, and x‐axes are community weighted N%. The Macro accent on the EFP indicates that the GPP_sat_ and GPP_sat.structure_ are the multiyear averages for each site. Bold *R* ^2^ and star symbols are for the relationships with ecosystem photosynthetic capacity estimates using PAR (GPP_sat_). Nonbold *R* ^2^ and round points are for the relationship with ecosystem photosynthetic capacity estimates using APAR (GPP_sat.structure_). The colors dark blue, light blue, dark green, light green, orange and yellow represent evergreen needle leaf forest, evergreen broad leaf forest, deciduous broad leaf forest, grassland, closed shrub‐land, and cropland as the plant functional types of the sites, respectively

Using a linear relationship, the N% based on data from the TRY database explained 27% of the variance of site averaged GPP_sat_ (20% of GPP_sat.structure_) (Figure 4a, Table 2). N% derived from TRY and in situ were strongly correlated (Fig. S5), and the *R* ^2^ of the relationship between N% and GPP_sat_, and GPP_sat.structure_ improved from 0.27 to 0.39 and from 0.20 to 0.32, respectively, when in situ N% was used (Figure 4b, Table 2). In addition, site averaged estimates of GPP_sat_ and GPP_sat.structure_ were replaced by GPP_sat_ and GPP_sat.structure_ from the years of in situ sampling *R* ^2^ increased to 0.50 and 0.37, respectively (Figure 4c, Table 2). The fit is even better when a nonlinear fit was used for Figure 4a,b (distance correlation increased from 0.56 to 0.73 for GPP_sat_ and from 0.47 to 0.63 for GPP_sat.structure,_ See also Fig. S6). An ANCOVA test revealed that the relationship between ecosystem photosynthetic capacity and N% was significantly different between the levels of synchronization when GPP_sat_ (significantly different in slope and intercept, *p *<* *.01) or GPP_sat.structure_ (only significantly different intercept, *p *<* *.05) was used to characterize ecosystem photosynthetic capacity. Similar improvements of the relationship of CWM traits to GPP_sat_ and GPP_sat.structure_ were realized using other plant traits and synchronizing the plant traits with the ecosystem photosynthetic capacity estimates in time and space (Fig. S2). We also tested whether the improvement of this relationship was due to random effects. To do this, we randomly resampled the annual photosynthetic capacity (specifically GPP_sat_ and GPP_sat.structure_) to test whether the use of corresponding years statistically improves the relationship or not. The results confirmed that the best fit was obtained when the N% and the photosynthetic capacity estimate match in time and space (Table S2).

**Table 2:** Statistics of the relationships shown in Figure 4

Ecosystem photosynthetic capacity	Model	Distance correlation	*R* ^2^	adj. *R* ^2^	Intercept ± *SE*	Slope ± *SE*	*p*	RRMSE	EF	*df*
GPP_sat_	N%	0.73	0.50	0.47	15.67 ± 3.51	7.25 ± 1.71	.0005	26.2	0.31	1 + 18
$\bar{GPP_{\mathrm{sat}}}$	N%	0.67	0.39	0.36	16.89 ± 3.95	6.57 ± 1.93	.003	29.09	0.18	1 + 18
$\bar{GPP_{\mathrm{sat}}}$	N%^TRY^	0.56	0.27	0.23	14.88 ± 5.74	8.55 ± 3.28	.018	30.65	0.09	1 + 18
${\mathrm{GPP}}_{\mathrm{sat}.\mathrm{structure}}$	N%	0.63	0.37	0.34	20.45 ± 5	7.62 ± 2.39	.005	30	0.10	1 + 17
$\bar{GPP_{\mathrm{sat}.\mathrm{structure}}}$	N%	0.58	0.32	0.28	21.18 ± 4.87	6.59 ± 2.33	.01	25.5	−0.15	1 + 17
$\bar{GPP_{\mathrm{sat}.\mathrm{structure}}}$	N%^TRY^	0.47	0.20	0.15	20.08 ± 7.01	8.07 ± 3.94	.06	26.1	−0.20	1 + 17

Ecosystem photosynthetic capacity estimates with macron accent are averaged over several years at each site and those without macron accent are from the year of leaf sampling. RRMSE and EF are estimated in a cross‐validation with leave‐one‐out mode and represents, relative root mean square error, and model efficiency, respectively. The number of FLUXNET sites that are used with GPP^sat^ are 20, but 19 of the sites have GPP_sat.structure_ available.

As species abundance information at the FLUXNET sites can be a relevant source of uncertainty, we also calculated site‐level species‐averaged N% without accounting for differences in abundance. The results of the *R* ^2^ decreased but only by about 0.05 (Fig. S7).

Part of the unexplained variance may be due to the fact that we used leaf level N%, while not accounting for differences in LAI. Indeed, although N% and LAI are highly correlated, the combination of N% and LAI led to a better explanation of the variability of GPP_sat,_ (adjusted *R* ^2^ = 0.56, *R* ^2^ = 0.64) than N% (*R* ^2^ = 0.50) or LAI (*R* ^2^ = 0.28) alone (Table 3—for 19 sites with available LAI).

**Table 3:** Relationships between N%, LAI, and GPP_sat_ tested

Variable	Model	Distance correlation	*R* ^2^	adj. *R* ^2^	Intercept ± *SE*	Slope ± *SE*	*p*	*df*	AIC
LAI	N%	0.70	0.48	0.45	0.34 ± 0.38	0.71 ± 0.18	.001	1 + 17	44
GPP_sat_	LAI	0.57	0.28	0.24	20.10 ± 4.03	5.43 ± 2.09	.01	1 + 17	138
GPP_sat_	N%	0.73	0.50	0.47	15.25 ± 3.79	7.41 ± 1.81	.0008	1 + 17	132
GPP_sat_	LAI + N%	0.71	0.50	0.44	14.96 ± 3.98	N% 6.78 ± 2.58LAI 0.87 ± 2.51	.004	2 + 16	134
GPP_sat_	N% + LAI + LAI:N%	—	0.64	0.56	0.74 ± 6.94	N% 15.22 ± 4.22LAI 10.33 ± 4.55N%:LAI −4.71 ± 1.98	.001	3 + 15	129

The GPP_sat_ is derived from the year at which the sampling of leaf N% was carried out. N% here is measured from in situ samples. LAI is the 90th percentile of the bimonthly LAI values retrieved from remote sensing and corresponds to the LAI of the sampling year as well (available for 19 sites).

### 3.3 Essential plant traits for ecosystem photosynthesis capacity

The variable selection analysis conducted with the stepwise regression using time‐space synchronized data of ecosystem photosynthetic capacity estimates and in situ measured plant traits and LAI showed that the variability of GPP_sat_ and GPP_sat.structure_ between sites is best explained by leaf C/N ratio and P_area_^2^ (considering AIC as the selection criteria). However, only C/N was a significant predictor for both of the ecosystem photosynthetic capacity estimates. The selected model explained 61% and 54% of the variance of GPP_sat_ and GPP_sat.structure_, respectively (Table 4).

**Table 4:** Results of the variable selection analyses conducted with a stepwise regression

Variable	Model	Distance correlation	*R* ^2^	adj. *R* ^2^	Intercept ± *SE*	Slope ± *SE*	*p*	*df*	AIC	EF
GPP_sat_	C/N + P_area_^2^	0.67	0.61	0.55	41.62 ± 3.01	C/N −0.39 ± 0.08P_area_^2^ 23.94 ± 16.20	.0009	2 + 15	119	0.18
GPP_sat.structure_	C/N + P_area_^2^	0.65	0.54	0.48	49.02 ± 4.07	C/N −0.48 ± 0.12P_area_^2^ 38.89 ± 22.22	.004	2 + 14	123	−0.28

The selected explanatory variables for GPPsat are C/N + P_area_^2^. The same variables are tested for GPP_sat_.structure as well. Subsets of sites are used because only 18 sites had these two traits available and GPP_sat_ and only 17 have the two traits and GPP_sat.structure_ measurements.

## 4 Discussion

### 4.1 Determining robust estimates of an EFP

We postulated that the IAV of ecosystem photosynthetic capacity at optimal growth conditions (e.g., at optimal light, temperature, and water availability) derived with the proposed methodology and in the absence of disturbances should be low, and we demonstrated that it is not strongly related to climate drivers (Fig. S8). Additionally, assuming that the variation of plant traits across years is relatively low, this would allow for coupling ecosystem photosynthetic capacity estimates at any year, or averaged over several years, to species traits collected at the respective site (typically sampled during peak growing season).

Based on these criteria, the use of the light response curves was suitable as it accounts for variation in radiation, which is one of the important parameters explaining variation in GPP (Van Dijk, Dolman, & Schulze, 2005). The estimation of the parameters using a moving window approach was also suitable because it accounts for variation in meteorological variables such as temperature and vapor pressure deficit. Among the parameters derived from the light response curve, *A*_max_ (or *A*_max.structure_) had the largest IAV and was therefore the least suitable estimator for ecosystem photosynthetic capacity. This may have several reasons: The response of GPP to PAR/APAR does not exhibit a clear saturation and still tends to increase at high PAR/APAR and reaches *A*_max_ outside the range of PAR/APAR measurements. Therefore, small changes in the slope at high PAR/APAR may cause large deviations in *A*_max_ (Gilmanov et al., 2003). In periods of the year when the PAR/APAR is not high, or the numbers of data points at high PAR is limited, the *A*_max_ parameter is poorly constrained. In this case, the fit can be affected by random flux uncertainty that scales with the magnitude of fluxes and is not easily constrainable (Richardson et al., 2012). GPP_sat_ or GPP_cum_ showed much smaller IAV, and therefore, we suggest the use GPP_sat_ or GPP_cum_ derived with PAR or APAR (Falge et al., 2001; Lasslop et al., 2010; Ruimy, Jarvis, Baldocchi, & Saugier, 1995) as more robust estimators of ecosystem photosynthetic capacity than *A*_max_. Our results also demonstrate that the use of higher percentiles (i.e., 90th) rather than the maximum for EFP extraction should be preferred as it was more robust to outliers.

### 4.2 Linking plant functional traits and EFP estimates

Ecosystem functional properties are whole‐ecosystem properties and thus depend on both ecosystem structure and function (Reichstein et al., 2014). As GPP depends on both the efficiency with which the absorbed energy is converted to chemical energy at leaf level (Monteith, 1972) and the canopy structure, GPP_sat_ variability ultimately depends on the variability of FAPAR (Reichstein et al., 2014). In this study, we accounted for this aspect using APAR in Equation (1) for the estimation of GPP_sat.structure_. APAR accounts for the seasonal and canopy structural (e.g., LAI) variability of the different ecosystems (Wang & Jarvis, 1990). In extreme combinations, it is possible for an ecosystem to maintain a high LAI but low N% and vice versa (Mcmurtrie et al., 2008; Fig. S9). However, due to the smoothing and reconstruction of time series of daily FAPAR from 16‐day data (e.g., Kandasamy, Baret, Verger, Neveux, & Weiss, 2013), and the spatial mismatch between satellite pixel and the eddy covariance footprint (Cescatti et al., 2012; Jung et al., 2008; Roman et al., 2009), the EFP estimates using APAR exhibited larger uncertainties that more likely is reflected in the higher IAV compared to using PAR. The FAPAR product that we used for our estimates has a high temporal resolution (16 days) but its spatial resolution (1 km) makes it uncertain; the footprints of FLUXNET sites are often smaller than a 1 km grid cell, and sites located in heterogeneous grid cells have higher uncertainties in FAPAR as a consequence (Cescatti et al., 2012). Nevertheless, the relationships of the estimates of photosynthetic capacity to plant traits were consistent, whether PAR or APAR was used. Our results also indicate the importance of accounting for canopy structure (Baldocchi & Meyers, 1998; Reich, 2012). The LAI‐N% interaction contributes to the explanatory power of the model for predicting GPP_sat_, as it shows how N% has an approximately linear relationship with GPP_sat_ (i.e., the GPP at light saturation without accounting for canopy structure) while the impact of LAI saturates.

A critical aspect when comparing leaf level attributes and EFPs is scaling these traits from leaf to canopy level. Based on the hypothesis that the dominant species are most adapted to their ambient environment (Vile, Shipley, & Garnier, 2006), also known as “dominance hypothesis” (Grime, 1998), we used CWM estimates of traits from dominant species at the sites. Here, we considered sites with different vegetation types and environments (e.g., climate), where differences between the locations and vegetation types are large enough to ignore intraspecific trait variability, this allows us to use averaged trait values from TRY database in this study and in likewise global scale analyses (see Albert, Grassein, Schurr, Vieilledent, & Violle, 2011).

### 4.3 Robustness of ecosystem photosynthetic capacity–plant trait relationship to relaxed time‐space synchrony of measurements

Here, we show that the general pattern of the relationship between ecosystem photosynthetic capacity and plant traits (slopes of the linear regression, Figure 4) is apparently independent using locally measured traits (in situ) or species mean values from the TRY database. In addition, the relationships are independent of whether all data corresponded to the same year or the ecosystem photosynthetic capacity represented the multiyear averages of ecosystem photosynthetic capacity we used (most cases, Fig. S2). However, we observed a strong degradation of the explained variance when the synchronization in time and space was relaxed. The predictive power of plant functional traits for ecosystem photosynthetic capacity substantially improved when variation of species abundance, intraspecific variability of plant traits, and interannual variability of ecosystem photosynthetic capacity were accounted for.

In part, this variability may be due to community species composition dynamics and competitive interactions that are partly triggered by disturbances or extreme environmental conditions. The study sites were not chosen to be in their late successional stage, and in the course of, for example, 10 years of flux measurements, species abundances can change and plant species can be replaced. Site history and aging of the ecosystems contributes to the variability of the plant traits (Becknell & Powers, 2014) and EFPs (e.g., Kutsch et al., 2009; Urbanski et al., 2007). This includes also the effect of fertilization on few sites, which could be one of the reasons why the in situ N% from the cropland and grasslands are very different from the mean N% from TRY. Plant traits also have a temporal variability, which can be due to plant development or changes in the environment (e.g., Mickelbart, 2010). Plant traits are responsible for the plastic response of an ecosystem to environmental changes and thus influence the interannual variability of ecosystem photosynthesis (Grassi, Vicinelli, Ponti, Cantoni, & Magnani, 2005; Ma, Baldocchi, Mambelli, & Dawson, 2010). Furthermore, it confirms that species signals of some traits, specifically leaf nutrients, are not strong enough (high trait variability) (Kazakou et al., 2014) and this contribute to the uncertainty observed when linking EFPs and trait values derived from data bases. One way to account for intraspecific trait variation is to use trait observations from TRY that were reported from similar climatic conditions to the FLUXNET sites, or to predict intraspecific trait variation (Schrodt et al., 2015). These opportunities are promising for future work, but could not be used here due to data scarcity and insufficient prediction accuracy. It remains to be better understood how the intraspecific variation of plant traits in time contributes to the response of plant communities to hydrometeorological changes and thus how the interannual and long‐term variability of ecosystem photosynthetic capacity is mediated by dynamics of the vegetation (Reichstein et al., 2014). A promising approach to monitor long‐term variation of plant traits for different FLUXNET sites worldwide is novel remote sensing information (e.g., Asner & Martin, 2015; Asner, Martin, Anderson, & Knapp, 2015). But the contribution of physiological vs. structural information in the remote sensing signals needs to be better understood (e.g., Homolova, Maenovsky, Clevers, Garcia‐Santos, & Schaeprnan, 2013; Wong & Gamon, 2015). The common protocols developed in initiatives like ICOS—integrated carbon observation system (https://www.icos-ri.eu/) and NEON—national ecological observatory network (http://www.neoninc.org/) might help to overcome such limitations.

### 4.4 Identifying plant traits determining ecosystem photosynthetic capacity

We considered leaf traits relevant for photosynthesis and used a data‐driven exploratory approach with all combinations of the selected leaf traits, mining for possible functional relationship between photosynthetic capacity and foliar traits (Golub, 2010). Our results are in line with other studies conducted at the leaf scale showing that C, N, and P stoichiometry have a complimentary role in explaining photosynthetic capacity (Perez‐Priego et al., 2015; Sardans & Penuelas, 2013; Walker et al., 2014). While C has low variation during the growing season (e.g., Jayasekera & Schleser, 1991; Kattge et al., 2011; Ma et al., 2010), N is the main factor driving the C/N ratio and influencing photosynthesis (see also Rong et al., 2015). The N% is related to the chlorophyll content (e.g., Houborg, Cescatti, Migliavacca, & Kustas, 2013) and to the amount of Ribulose‐1,5‐bisphosphate carboxylase/oxygenase enzymes that ultimately controls the photosynthetic rates and carbon uptake (Evans, 1989; Kattge et al., 2009). Several studies have also shown this link at the ecosystem level (Kergoat et al., 2008; Ollinger et al., 2008; Reich, 2012). P is found in adenosine triphosphate molecules (ATP) and nucleotides of nicotinamide adenine dinucleotide phosphate (NADP), which are involved in carbon fixation reactions. Several hypotheses connect the stoichiometry of leaves with optimum photosynthetic capacity and growth (e.g., growth rate hypothesis) (Elser, O'brien, Dobberfuhl, & Dowling, 2000; Sterner & Elser,^2002^). In particular, the N/P ratio is related to photosynthetic capacity via the connection between the allocation of P into P‐rich ribosomal RNA and of N to protein synthesis (Hessen, Jensen, Kyle, & Elser, 2007). As P is also used in carbon fixation as N, it influences the nitrogen‐photosynthesis relationship by constraining the response of photosynthesis to N when P is low (Reich et al., 2009; Walker et al., 2014). However, more data are needed to build robust models that predict ecosystem photosynthetic capacity directly from plant functional traits and stoichiometry. Currently, no consensus exists on which traits are most important to be measured at the sites in order to monitor the effect of plants on ecosystem functioning in response to their environment. Trait‐ecosystem functioning studies with more data are needed to allow for robust conclusion on a suit of traits in this regard.

In conclusion, to quantitatively evaluate the link between ecosystem photosynthetic capacity and plant traits to improve predictions of ecosystem carbon uptake, continuous observations of species composition and plant traits at FLUXNET sites can be the key. We showed that currently the evaluation is limited by the scarcity of observations of both species composition and traits. We therefore suggest systematic sampling of plant traits, species abundance, and auxiliary data for upscaling traits at FLUXNET sites in parallel to flux measurements. In addition, remote sensing can be a solution in the future to acquire canopy level traits, circumventing upscaling issues of in situ measurements and may contribute to better detection of temporal and spatial variation of ecosystem level plant traits in synchrony with ecosystem photosynthetic capacity.

## Conflict of Interest

None declared.
