# Supplementary material for: Potential and limitations of inferring ecosystem photosynthetic capacity from leaf functional traits
Source: Ecol Evol. 2016 Sep 22;6(20):7352–66. doi: 10.1002/ece3.2479 (PMC5513259; doi:10.1002/ece3.2479)
Supplement: Supplementary file 1 [file ECE3-6-7352-s001.docx]

SUPPORTING MATERIAL

Table S1 Summary of Pearson correlation coefficient between the different estimates of ecosystem photosynthetic capacity.

|  | GPP_sat_ | GPP_sat.structure_ | A_max_ | A_max.structure_ | GPP_cum_ | GPP_cum.structure_ |
| --- | --- | --- | --- | --- | --- | --- |
| GPP_sat_ | 1 |  |  |  |  |  |
| GPP_sat.structure_ | 0.93 | 1 |  |  |  |  |
| A_max_ | 0.82 | 0.90 | 1 |  |  |  |
| A_max.structure_ | 0.84 | 0.95 | 0.94 | 1 |  |  |
| GPP_cum_ | 0.97 | 0.85 | 0.71 | 0.73 | 1 |  |
| GPP_cum.structure_ | 0.95 | 0.95 | 0.86 | 0.86 | 0.90 | 1 |

**Table S2 In order to compare Fig. 4b with Fig. 4c in respect to random effects (whether the relationship in Fig. 4c is just by chance better than Fig. 4b), we performed a bootstrapping test. For the relationship in Fig. 4b prior to estimating the mean GPP_sat_ (or GPP_sat.structure_) of the sites, we sampled randomly (with replacement) for each site the annual GPP_sat_ (also GPP_sat.structure_) and then estimated the mean over the years. This was done 100 times and at each step the linear regression of the model was tested for R^2^ and *p-value*. In none of the cases the fit was better than the one in Fig. 4c when GPP_sat_ was used (time and space matched data). Using GPP_sat.structure_ only 1% of the random site-year combination had an R^2^ higher than the one in Fg.4c (0.37) with a *p-value* < 0.05 and a positive slope. Below are the summary of the results from the 100 random fit of Fig. 4b.**

|  | R^2^ | *p.value* | EFP estimate |
| --- | --- | --- | --- |
| Min. | 0.2683 | 0.002288 | **GPP_sat_** |
| 1^st^ Qu. | 0.2845 | 0.004134 |  |
| Median | 0.3315 | 0.007891 |  |
| Mean | 0.3309 | 0.009602 |  |
| 3^rd^ Qu. | 0.3745 | 0.015435 |  |
| Max. | 0.4118 | 0.019314 |  |
| Min. | 0.2799 | 0.002061 | **GPP_sat.structure_** |
| 1^st^ Qu. | 0.2967 | 0.007480 |  |
| Median | 0.3345 | 0.009494 |  |
| Mean | 0.3320 | 0.011045 |  |
| 3^rd^ Qu. | 0.3514 | 0.015927 |  |
| Max. | 0.4369 | 0.019853 |  |

Figure S1 Time series of daily GPP_sat_. Data filtering using the R^2^ of the model fit shows that only GPP_sat_ during growing season will be selected (colored in dark green). The related model fit R^2^ of the filtered data is shown in red stars. The example is made for two sites with two different plant functional types. ENF is ever green needle leaved forest and DBF is for deciduous broad leaved forest.


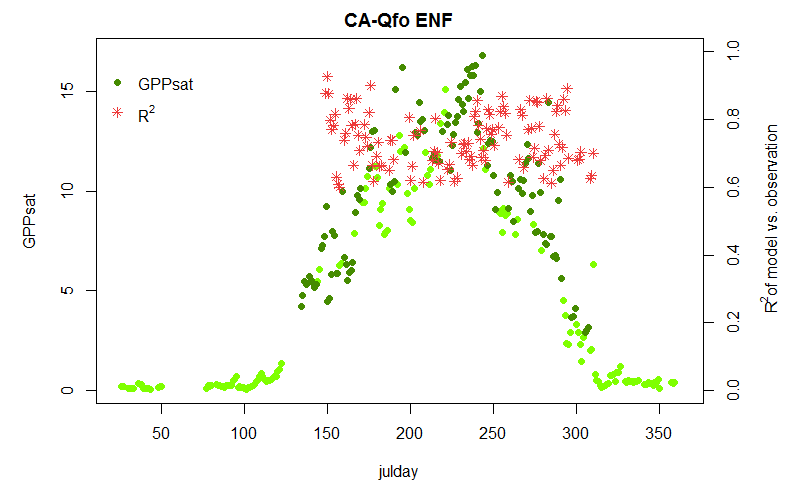

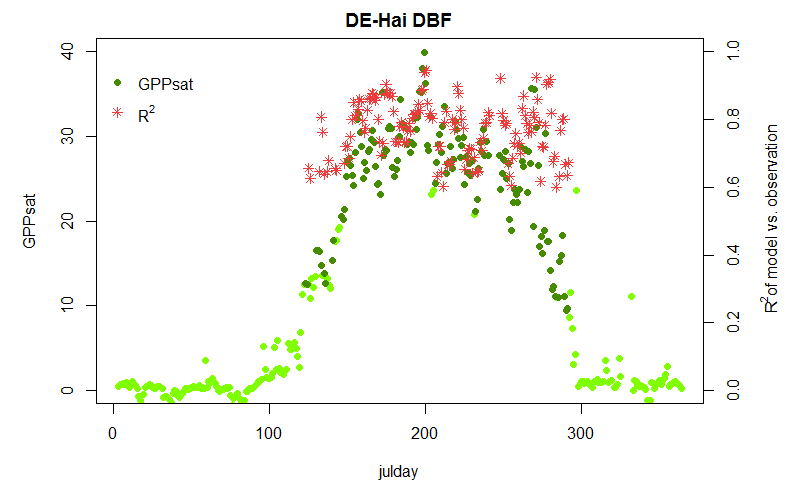


Figure S2 a) Relationship between GPP_sat_ and GPP_sat.structure_ extracted from La Thuile and the trait from TRY. b) GPP_sat_ and GPP_sat.structure_ from La Thuile and the trait from *in-situ* measurements. c) GPP_sat_ and GPP_sat.structure_ derived from the same year of the trait sampling and the trait from *in-situ* measurements. The Macro accent on the EFP indicates that the GPP_sat_ and GPP_sat.structure_ are the multi-year averages for each site. The traits are all community weighted averaged. The adjusted R^2^ of the relationship is shown in the figures in case there was a significant relationship (0.05>p-value). Bold R^2^ and star symbols are for the relationships with GPP_sat_ as the EFP estimate. Non-bold R^2^ and round points are for the relationship with GPP_sat.structure_ as the EFP estimate. The colors dark blue, light blue, dark green, light green, orange and yellow represent evergreen needle leaf forest, evergreen broad leaf forest, deciduous broad leaf forest, grassland, closed shrub-land and cropland as the plant functional types of the sites, respectively.


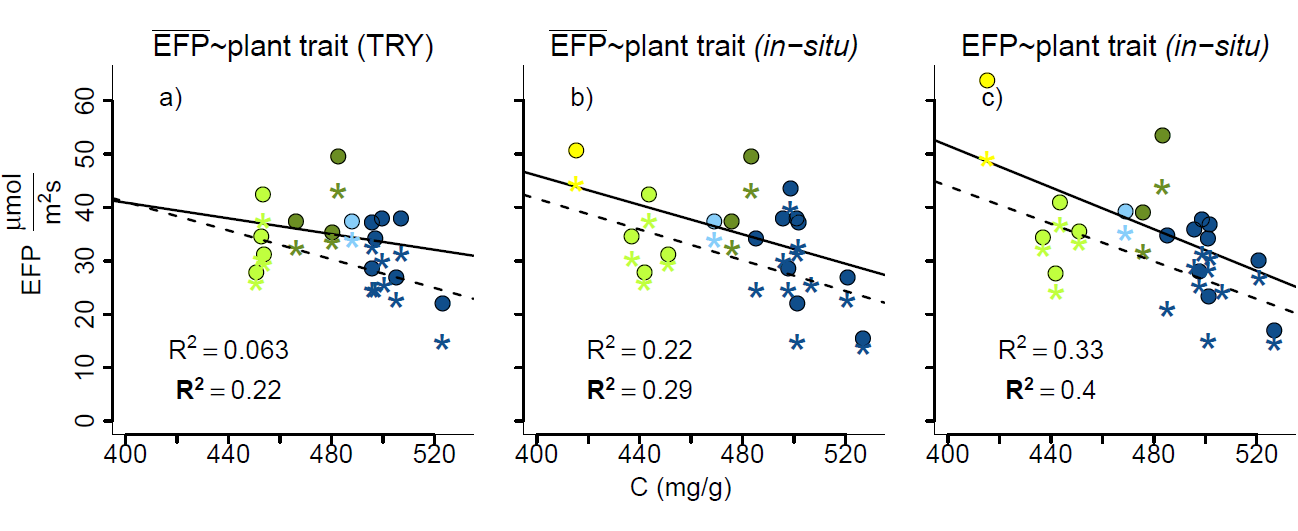

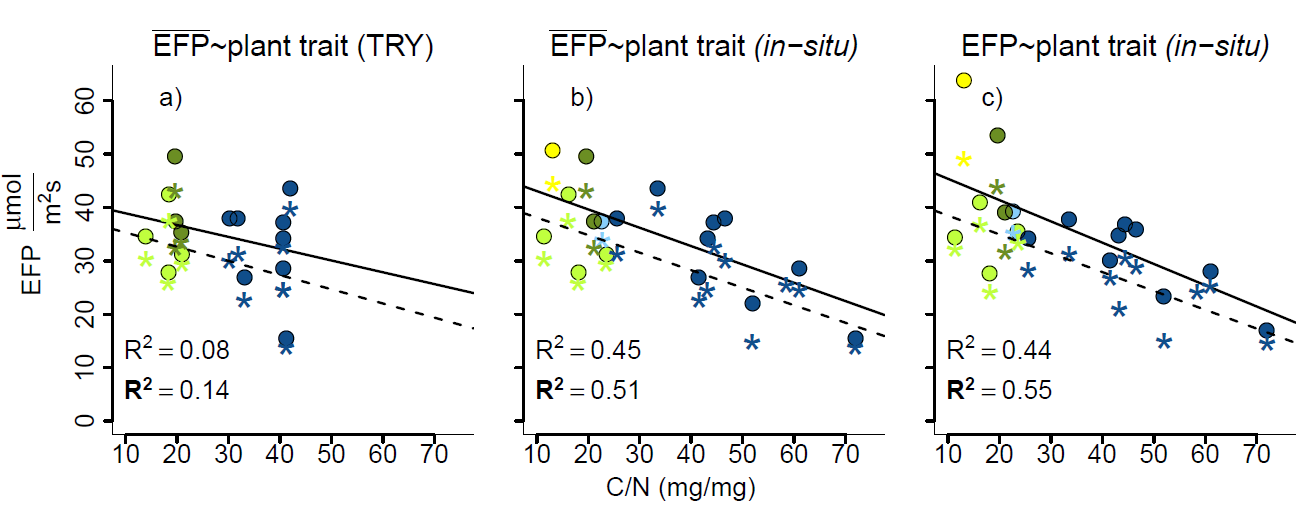

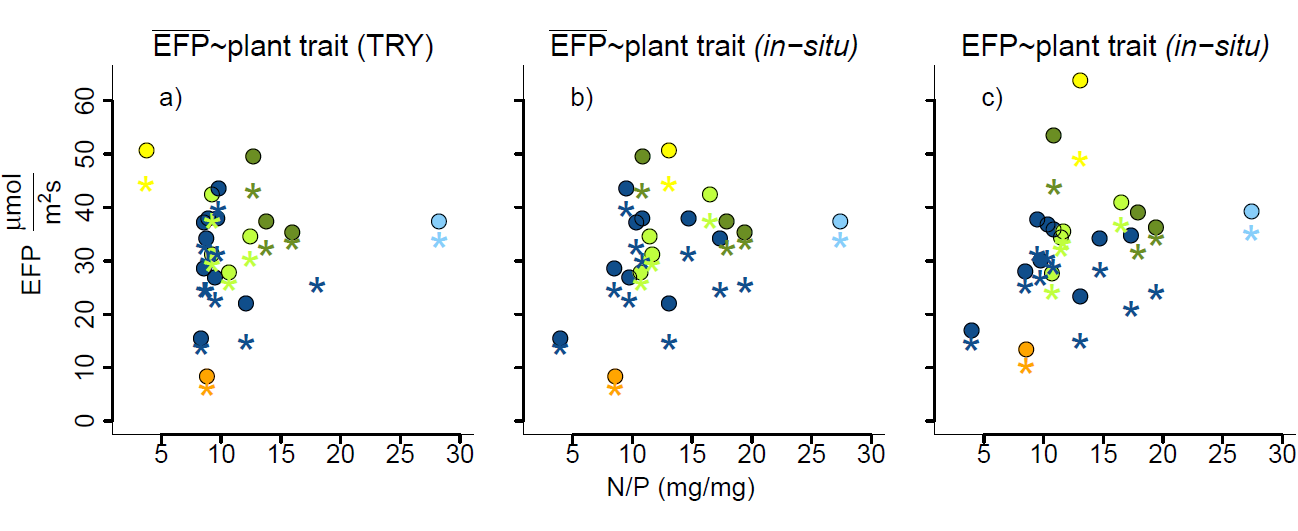

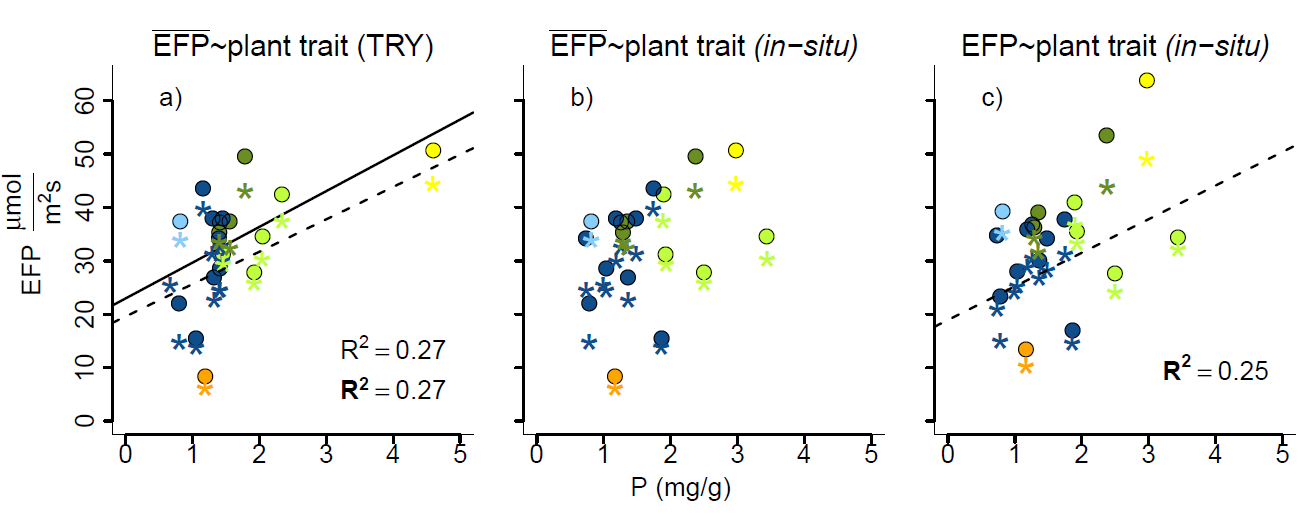

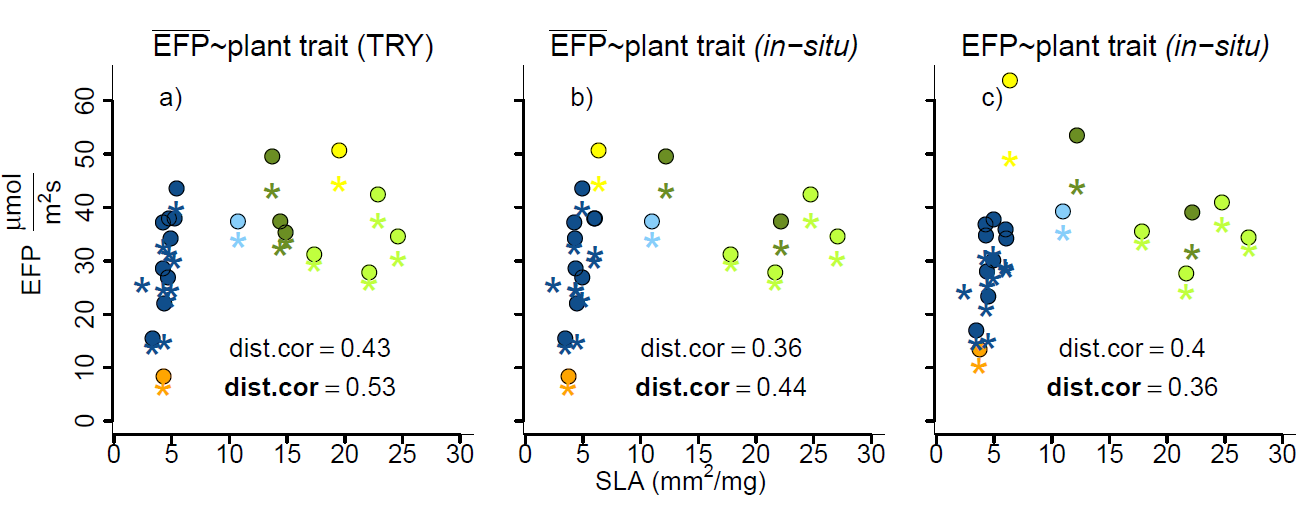


Figure S3 Boxplots of annual GPP_sat_ values derived from the La Thuile database for each FLUXNET site. The red point denotes GPP_sat_ values of the 2003 year were a heat wave happened in Europe. For some European sites that year 2003 is removed already due to prepossessing of GPP_sat_ estimates.


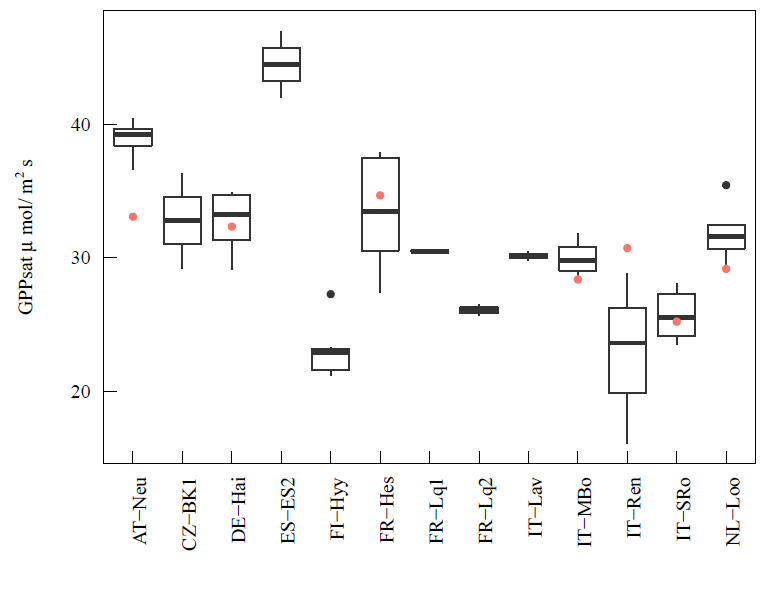


**Figure S4 Relationship between a) GPP_sat_ and GPP_sat.structure_ extracted from La Thuile and N% from TRY, b) GPP_sat_ and GPP_sat.structure_ from La Thuile and N% *in-situ*. The Macro accent on the EFP indicates that the GPPsat and GPP_sat.structure_ are the multi-year averages for each site. Here the 2003 year related to the heat wave was removed for European sites before using the averages.**


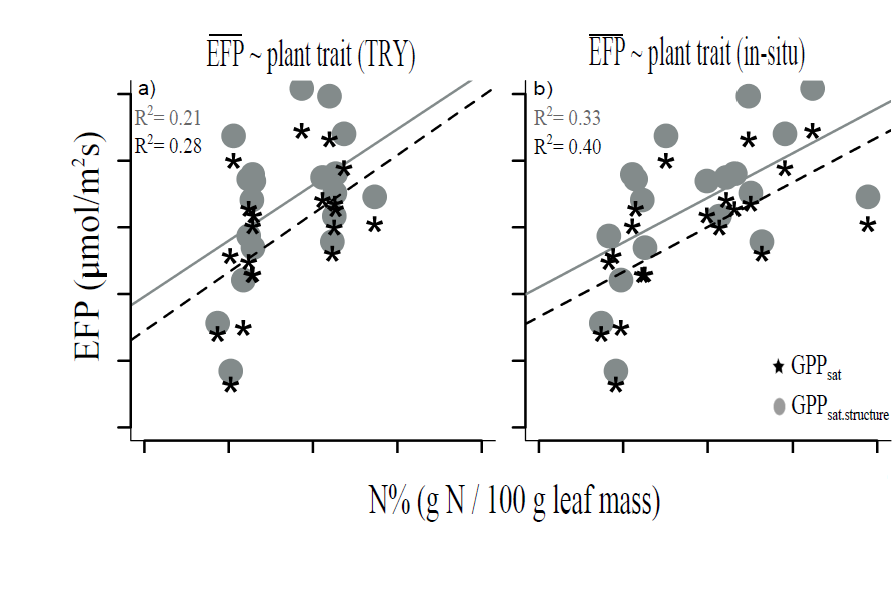


Figure S5 Summary of the fit between the *in-situ* measured and database derived community weighted mean of the plant traits. X-axes are plant traits from TRY and Y-axes are *in-situ* plant traits. The numbers on the left upper corner are the Pearson correlation coefficients between the two sources of plant traits.


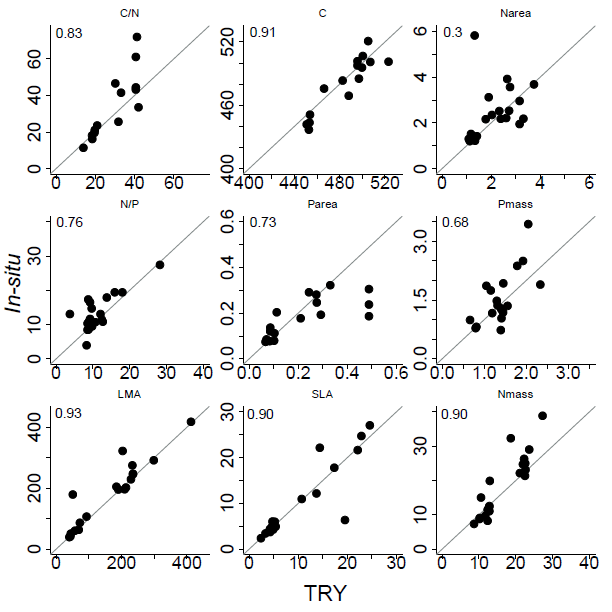


Figure S6 From left to right relationship between GPP_sat_ and GPP_sat.structure_ extracted from La Thuile and N% from TRY. GPP_sat_ and GPP_sat.structure_ from La Thuile and N% *in-situ*. GPP_sat_ and GPP_sat.structure_ derived from the same year of the trait sampling and N% *in-situ*. The Macro accent on the EFP indicates that the GPP_sat_ and GPP_sat.structure_ are the multi-year averages for each site. N% is the abundance weighted gram nitrogen per 100 gram leaf mass. The adjusted R^2^ of the relationship is shown in the figures. Bold R^2^ and star symbols are for the relationships with GPP_sat_ as the EFP estimate. Non-bold R^2^ and round points are for the relationship with GPP_sat.structure_ as the EFP estimate. The colors dark blue, light blue, dark green, light green, orange and yellow represent evergreen needle leaf forest, evergreen broad leaf forest, deciduous broad leaf forest, grassland, closed shrub-land and cropland as the plant functional types of the sites, respectively.
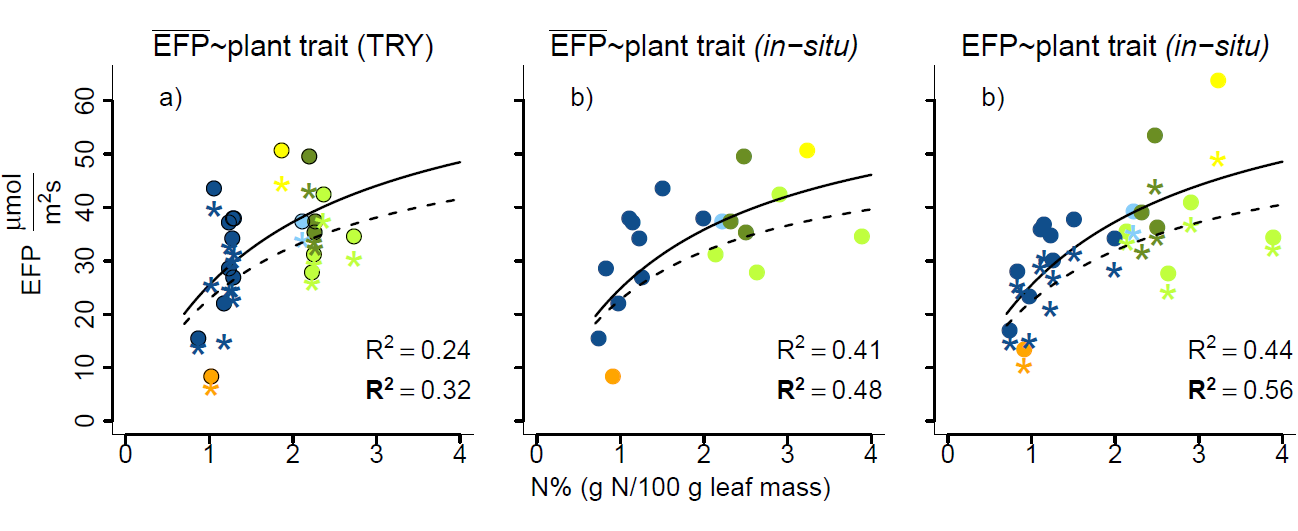


Figure S7 From left to right Relationship between GPP_sat_ and GPP_sat.structure_ extracted from La Thuile and N% from TRY. GPP_sat_ and GPP_sat.structure_ from La Thuile and N% *in-situ*. GPP_sat_ and GPP_sat.structure_ derived from the same year of the trait sampling and N% *in-situ*. The Macro accent on the EFP indicates that the GPP_sat_ and GPP_sat.structure_ are the multi-year averages for each site. N% is the average of species N% at each site. The adjusted R^2^ of the relationship is shown in the figures in case there was a significant relationship (0.05>p-value). Bold R^2^ and star symbols are for the relationships with GPP_sat_ as the EFP estimate. Non-bold R^2^ and round points are for the relationship with GPP_sat.structure_ as the EFP estimate. The colors dark blue, light blue, dark green, light green, orange and yellow represent evergreen needle leaf forest, evergreen broad leaf forest, deciduous broad leaf forest, grassland, closed shrub-land and cropland as the plant functional types of the sites, respectively.
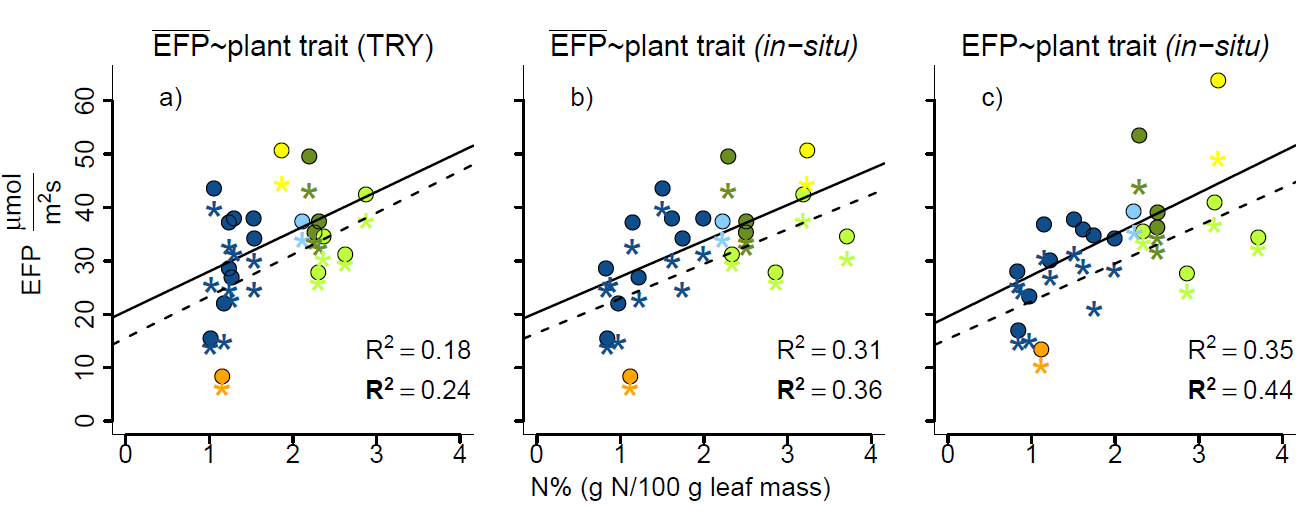


**Figure S8 The extraction of GPP_sat_ considers the optimal conditions and thereby less correlated to climate variables. The fact that GPP_sat_ is the potential GPP at light saturation overcomes the direct effects of climate as well. The two figures below indicate that the difference between sites for GPP_sat_ is not related to the mean precipitation and only slightly related to air temperature of the sites. Both climate variables were estimated during the growing season. In a and b the link between the annual average air temperature and cumulative precipitation is shown with annual GPP_sat_. In c and d the link between mean annual temperature (MAT) and mean annual precipitation (MAP) with the site averaged GPP_sat_ is shown.**

**
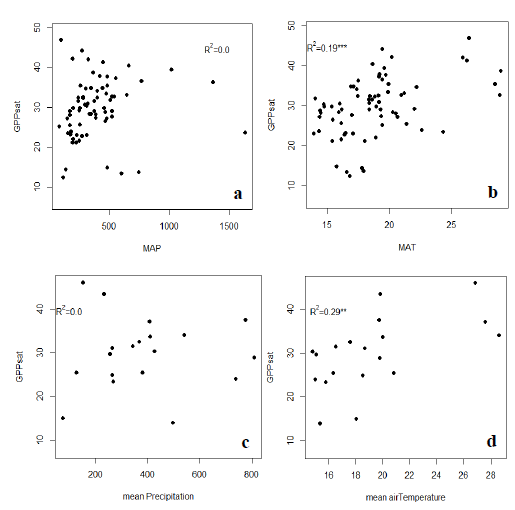
**

Figure S9 Relationship between N% (here total canopy nitrogen content divided by LAI) and photosynthetic capacity Simulations of GPP2000 were done using De Pury & Farquhar (1997) model, based on the combination of Farquhar photosynthesis model (Farquhar *et al.,* 1980) with the two-leaf big-leaf presentation of the canopy radiative transfer. Simulations were done with a given leaf temperature, prescribed Ci (25 Pa), a diffuse fraction of 20% and a solar angle of 65° and turning off daytime mitochondrial respiration. Vcmax at 25°C in the model depends on leaf nitrogen content (N%) – forbs parameterisation from Wohlfahrt *et al.,* (1999; Fig. 3a) were used. LAI simulations vary from 0.5-8 m2/m2 and N% of the uppermost leaves varying from 1.5-4.5%.


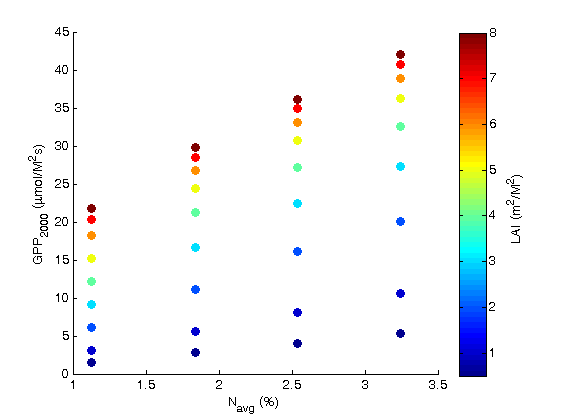


**REFERENCES:**

De Pury DGG, Farquhar GD (1997) Simple scaling of photosynthesis from leaves to canopies without the errors of big-leaf models. Plant Cell and Environment, 20, 537-557.

Farquhar GD, Caemmerer SV, Berry JA (1980) A Biochemical-Model of Photosynthetic Co2 Assimilation in Leaves of C-3 Species. Planta, 149, 78-90.

Wohlfahrt G, Bahn M, Haubner E et al. (1999) Inter-specific variation of the biochemical limitation to photosynthesis and related leaf traits of 30 species from mountain grassland ecosystems under different land use. Plant Cell and Environment, 22, 1281-1296.
