# Supplementary material for: Potential and limitations of inferring ecosystem photosynthetic capacity from leaf functional traits
Source: Ecol Evol. 2016 Sep 22;6(20):7352–66. doi: 10.1002/ece3.2479 (PMC5513259; doi:10.1002/ece3.2479)
Supplement: Supplementary file 5 [file ECE3-6-7352-s005.docx]

**SUPPLEMENTARY DATA-TABLES**

**InSitu_Traits**

Table provides the species sampled at the each site with the measured traits. Mean.trait is the averaged trait value, whereas Inv.No is the number of individuals that were sampled used to estimate the average trait value and the standard deviation of the trait values (SD.trait). DOY is the day of the year when the sampling was done. For more information please contact Martine Janet van de Weg (marjan@marjanvandeweg.com).

**CWMtraits_EFPs**

Community weighted traits and ecosystem photosynthetic capacity estimates for the 20 sites (used in the analyses of the link between plant functional traits and ecosystem photosynthetic capacity). CWM at the beginning of the column names means that the values are community weighted means of in-situ data and CWMT is for community weighted means of traits from TRY database.

**SpeciesComposition**

Species names and abundance of the FLUXNET sites. Only for BR-Sa1 we did not have access to the species abundance and therefore equal abundances for the species are considered.
